# Caffeine use in preterm neonates: national insights into Turkish NICU practices

**DOI:** 10.3389/fped.2025.1492716

**Published:** 2025-02-27

**Authors:** Sezin Unal, Serdar Beken, Deniz Anuk Ince, Ozden Turan, Ayse Korkmaz Toygar, Ayse Ecevit, Abdullah Baris Akcan, Mustafa Ali Akın, Selma Aktas, Nukhet Aladag Ciftdemir, Emel Altuncu, Huseyin Altunhan, Baran Cengiz Arcagok, Didem Armangil, Esra Arun Ozer, Banu Aydın, Handan Bezirganoglu, Leyla Bilgin, Erhan Calısıcı, Sebnem Calkavur, Kıymet Celik, Yalcın Celik, Bilin Cetinkaya, Merih Cetinkaya, Atalay Demirel, Gamze Demirel, Nazan Neslihan Dogan, Pelin Doğan, Mehtap Durukan, Defne Engur, Tugba Erener Ercan, Zeynel Gokmen, Ipek Guney Varal, Selvi Gulası, Ayla Gunlemez, Tugba Gursoy, Handan Hakyemez Toptan, Serif Hamitoğlu, Fatih Isleyen, Irem Iyigun, Sebnem Kader, Dilek Kahvecioğlu, Gozdem Kaykı, Murat Kostu, Dilek Kurnaz, Tural Mammadalıyev, Ilke Mungan Akin, Nejat Narlı, Emel Okulu, Nilufer Okur, Ozgur Olukman, Fahri Ovalı, Beyza Ozcan, Ahmet Ozdemir, Ozmert Ozdemir, Hilal Ozkan, Gonca Sandal, Dilek Sarıcı, Cansu Sivrikaya, Betul Siyah Bilgin, Saime Sundus, Ozge Surmeli Onay, Huseyin Simsek, Umit Ayse Tandırcıoğlu, Sema Tanrıverdi, Kadir Serafettin Tekgunduz, Demet Terek, Gaffari Tunc, Turan Tunc, Ercan Tutak, Eda Tufekcioğlu, Funda Tuzun Erdogan, Ersin Ulu, Dilek Ulubas Isik, Nurdan Uras, Sait Ilker Uslu, Irem Unal, Fatma Hilal Yılmaz, Ariorad Moniri

**Affiliations:** ^1^ Division of Neonatology, Department of Pediatrics, Baskent University Faculty of Medicine, Ankara, Türkiye; ^2^ Division of Neonatology, Department of Pediatrics, Acıbadem Mehmet Ali Aydınlar University Faculty of Medicine, Atakent Hospital, İstanbul, Türkiye; ^3^ Division of Neonatology, Department of Pediatrics, Aydın Adnan Menderes University Faculty of Medicine, Aydın, Türkiye; ^4^ Division of Neonatology, Department of Pediatrics, On Dokuz Mayıs University Faculty of Medicine, Samsun, Türkiye; ^5^ Division of Neonatology, Department of Pediatrics, Acıbadem Mehmet Ali Aydınlar University Faculty of Medicine, Maslak Hospital, İstanbul, Türkiye; ^6^ Division of Neonatology, Department of Pediatrics, Trakya University Faculty of Medicine, Edirne, Türkiye; ^7^ Division of Neonatology, Department of Pediatrics, University of Health Sciences, Kartal Dr Lutfi Kırdar City Hospital, İstanbul, Türkiye; ^8^ Division of Neonatology, Department of Pediatrics, Necmettin Erbakan University Faculty of Medicine, Konya, Türkiye; ^9^ Division of Neonatology, Department of Pediatrics, Acıbadem Mehmet Ali Aydınlar University Faculty of Medicine, Altunizade Hospital, İstanbul, Türkiye; ^10^ Division of Neonatology, Department of Pediatrics, Koru Ankara Hospital, Ankara, Türkiye; ^11^ Division of Neonatology, Department of Pediatrics, İzmir Tınaztepe University Faculty of Medicine, İzmir, Türkiye; ^12^ Division of Neonatology, Department of Pediatrics, Lokman Hekim University Faculty of Medicine, Ankara, Türkiye; ^13^ Division of Neonatology, Department of Pediatrics, Trabzon Kanuni Training and Research Hospital, Trabzon, Türkiye; ^14^ Division of Neonatology, Department of Pediatrics, İstanbul University İstanbul Faculty of Medicine, İstanbul, Türkiye; ^15^ Division of Neonatology, Department of Pediatrics, University of Health Sciences Gulhane Faculty of Medicine, Gulhane Training and Research Hospital, Ankara, Türkiye; ^16^ Division of Neonatology, Department of Pediatrics, University of Health Sciences İzmir Dr. Behcet Uz Pediatric and Pediatric Surgery Training and Research Hospital, İzmir, Türkiye; ^17^ Division of Neonatology, Department of Pediatrics, Akdeniz University Faculty of Medicine, Antalya, Türkiye; ^18^ Division of Neonatology, Department of Pediatrics, Mersin University Faculty of Medicine, Mersin, Türkiye; ^19^ Division of Neonatology, Department of Pediatrics, Baskent University Faculty of Medicine, Adana Dr. Turgut Noyan Training and Research Center, Adana, Türkiye; ^20^ Division of Neonatology, Department of Pediatrics, University of Health Sciences, Basaksehir Cam Sakura City Hospital, İstanbul, Türkiye; ^21^ Division of Neonatology, Department of Pediatrics, Acıbadem Mehmet Ali Aydınlar University Faculty of Medicine, Acıbadem Kadıkoy Sinasi Can Hospital, İstanbul, Türkiye; ^22^ Division of Neonatology, Department of Pediatrics, Koc University Faculty of Medicine, Amerikan Hospital, İstanbul, Türkiye; ^23^ Division of Neonatology, Department of Pediatrics, University of Health Sciences Bakırkoy Dr. Sadi Konuk Training and Research Hospital, İstanbul, Türkiye; ^24^ Division of Neonatology, Department of Pediatrics, Acıbadem Mehmet Ali Aydınlar University Faculty of Medicine, Acıbadem Atasehir Hospital, İstanbul, Türkiye; ^25^ Division of Neonatology, Department of Pediatrics, Mardin Training and Research Hospital, Mardin, Türkiye; ^26^ Division of Neonatology, Department of Pediatrics, University of Health Sciences Tepecik Training and Research Hospital, İzmir, Türkiye; ^27^ Division of Neonatology, Department of Pediatrics, Maltepe University Faculty of Medicine, İstanbul, Türkiye; ^28^ Division of Neonatology, Department of Pediatrics, Baskent University Faculty of Medicine, Konya Training and Research Hospital, Konya, Türkiye; ^29^ Division of Neonatology, Department of Pediatrics, University of Health Sciences Bursa Yuksek İhtisas Training and Research Hospital, Bursa, Türkiye; ^30^ Division of Neonatology, Department of Pediatrics, University of Health Sciences Adana Sehir Hospital, Adana, Türkiye; ^31^ Division of Neonatology, Department of Pediatrics, Kocaeli University Faculty of Medicine, Kocaeli, Türkiye; ^32^ Division of Neonatology, Department of Pediatrics, Koc University Faculty of Medicine, Koc University Hospital, İstanbul, Türkiye; ^33^ Division of Neonatology, Department of Pediatrics, Marmara University Faculty of Medicine Pendik Training and Research Hospital, İstanbul, Türkiye; ^34^ Division of Neonatology, Department of Pediatrics, Bursa Medicana Hospital, Bursa, Türkiye; ^35^ Division of Neonatology, Department of Pediatrics, Sanlıurfa Training and Research Hospital, Urfa, Türkiye; ^36^ Division of Neonatology, Department of Pediatrics, Ordu University Training and Research Hospital, Ordu, Türkiye; ^37^ Division of Neonatology, Department of Pediatrics, Kırklareli Training and Research Hospital, Kırlareli, Türkiye; ^38^ Division of Neonatology, Department of Pediatrics, University of Health Sciences Ankara Training and Research Hospital, Ankara, Türkiye; ^39^ Division of Neonatology, Department of Pediatrics, Hacettepe University Faculty of Medicine, Ankara, Türkiye; ^40^ Division of Neonatology, Department of Pediatrics, University of Health Sciences İstanbul Kanuni Sultan Suleyman Training and Research Hospital, İstanbul, Türkiye; ^41^ Division of Neonatology, Department of Pediatrics, İstanbul Haseki Training and Research Hospital, İstanbul, Türkiye; ^42^ Division of Neonatology, Department of Pediatrics, Gazı University Faculty of Medicine, Ankara, Türkiye; ^43^ Division of Neonatology, Department of Pediatrics, University of Health Sciences Umraniye Training and Research Hospital, İstanbul, Türkiye; ^44^ Division of Neonatology, Department of Pediatrics, Cukurova University Faculty of Medicine, Adana, Türkiye; ^45^ Division of Neonatology, Department of Pediatrics, Ankara University Faculty of Medicine, Ankara, Türkiye; ^46^ Division of Neonatology, Department of Pediatrics, University of Health Sciences Gazi Yasargil Training and Research Hospital, Diyarbakır, Türkiye; ^47^ Division of Neonatology, Department of Pediatrics, İzmir BakırCay University Faculty of Medicine, Ciğli Training and Research Hospital, İzmir, Türkiye; ^48^ Division of Neonatology, Department of Pediatrics, İstanbul Medeniyet University Goztepe Prof. Dr. Suleyman YalCın City Hospital, İstanbul, Türkiye; ^49^ Division of Neonatology, Department of Pediatrics, University of Health Sciences Konya City Hospital, Konya, Türkiye; ^50^ Division of Neonatology, Department of Pediatrics, University of Health Sciences Kayseri City Hospital, Kayseri, Türkiye; ^51^ Division of Neonatology, Department of Pediatrics, Pamukkale University Faculty of Medicine, Denizli, Türkiye; ^52^ Division of Neonatology, Department of Pediatrics, Uludağ University Faculty of Medicine, Bursa, Türkiye; ^53^ Division of Neonatology, Department of Pediatrics, Medipol University Faculty of Medicine, Medipol Mega Hospital, İstanbul, Türkiye; ^54^ Division of Neonatology, Department of Pediatrics, University of Health Sciences Ankara Ataturk Sanatoryum Training and Research Hospital, Ankara, Türkiye; ^55^ Division of Neonatology, Department of Pediatrics, Hatay Training and Research Hospital, Hatay, Türkiye; ^56^ Division of Neonatology, Department of Pediatrics, University of Health Sciences Ankara Bilkent Sehir Hospital, Ankara, Türkiye; ^57^ Division of Neonatology, Department of Pediatrics, SelCuk University Faculty of Medicine, Konya, Türkiye; ^58^ Division of Neonatology, Department of Pediatrics, Eskisehir Osmangazi University Faculty of Medicine, Eskisehir, Türkiye; ^59^ Division of Neonatology, Department of Pediatrics, University of Health Sciences Mersin City Hospital, Mersin, Türkiye; ^60^ Division of Neonatology, Department of Pediatrics, Kırıkkale University Faculty of Medicine, Kırıkkale, Türkiye; ^61^ Division of Neonatology, Department of Pediatrics, Manisa Celal Bayar University Faculty of Medicine, Manisa, Türkiye; ^62^ Division of Neonatology, Department of Pediatrics, Ataturk University Faculty of Medicine, Erzurum, Türkiye; ^63^ Division of Neonatology, Department of Pediatrics, Ege University Faculty of Medicine, İzmir, Türkiye; ^64^ Division of Neonatology, Department of Pediatrics, Sivas Cumhuriyet University Faculty of Medicine, Sivas, Türkiye; ^65^ Division of Neonatology, Department of Pediatrics, Memorial Atasehir Hospital, İstanbul, Türkiye; ^66^ Division of Neonatology, Department of Pediatrics, University of Health Sciences Prof. Dr. Cemil Tascıoğlu City Hospital, İstanbul, Türkiye; ^67^ Division of Neonatology, Department of Pediatrics, University of Health Sciences Ankara Etlik City Hospital, Ankara, Türkiye; ^68^ Division of Neonatology, Department of Pediatrics, Dokuz Eylul University Faculty of Medicine, İzmir, Türkiye; ^69^ Division of Neonatology, Department of Pediatrics, İstanbul Universitesi- Cerrahpasa, Cerrahpasa Faculty of Medicine, İstanbul, Türkiye; ^70^ Division of Neonatology, Department of Pediatrics, University of Health Sciences Etlik Zubeyde Hanım Maternal Health Training and Research Hospital, Ankara, Türkiye; ^71^ Division of Neonatology, Department of Pediatrics, İstinye University Bahcesehir Liv Hospital, İstanbul, Türkiye; ^72^ Division of Neonatology, Department of Pediatrics, Demiroğlu Bilim University İstanbul Florence Nightingale Hospital, İstanbul, Türkiye; ^73^ Division of Neonatology, Department of Pediatrics, Sehit Prof. Dr. İlhan Varank Sancaktepe Training and Research Hospital, İstanbul, Türkiye; ^74^ Division of Neonatology, Department of Pediatrics, Dr. Ali Kemal Belviranli Obstetrics and Gynecology Hospital, Konya, Türkiye; ^75^ Department of Biostatistics, Acıbadem Mehmet Ali Aydınlar University Faculty of Medicine, İstanbul, Türkiye

**Keywords:** caffeine, premature neonate, apnea of prematurity, neonatal intensive care unit, guideline

## Abstract

**Objective:**

Caffeine is a proven medication used for the prevention and treatment of apnea in premature infants, offering both short- and long-term benefits. International guidelines provide a range of recommendations regarding the preterm population eligible for caffeine prophylaxis, including the timing, dosage, and duration of treatment. Our national guidelines, published prior to the most recent updates of the international guidelines, recommend the use of caffeine citrate starting from the first day after delivery for preterm infants with a gestational age of <28 weeks. For infants up to 32 weeks, if positive pressure ventilation is required, the decision should be made on an individual basis. This study aims to describe the variability in caffeine usage across neonatal intensive care units in our country.

**Methods:**

An online survey was sent to neonatologist who are members of the Turkish Neonatology Society to describe the variability in caffeine usage in neonatal intensive care units in our country.

**Results:**

We collected responses from 74 units. Prophylactic caffeine usage was observed as; GA ≤27^6/7^: 98.6%, GA 28^0/7^–28^6/7^: 89.0%, GA 29^0/7^–29^6/7^: 75.3%, GA 30^0/7^–31^6/7^: 53.4%. 62.2% of units reported administering loading dose within the first two hours. The initial maintenance dose was 5 mg/kg in 64.8% of units, 10 mg/kg in 32.4% of units, and intermediate dose in 5.3% of units. 47.3% of units reported no routine dose adjustment. The postmenstrual age that caffeine treatment was stopped was found to be 34 (min-max; 32–36) weeks for infants without apnea and respiratory support, 36 (min-max; 34–52) weeks for infants without apnea but any respiratory support. The time to discharge after treatment cessation was found as; 1–4 days: 37.8%, 5–7 days: 68.9%. Among the 56 units with multiple responsible physicians, 32.1% reported intra-unit variations.

**Conclusion:**

The significant differences in caffeine usage characteristics between and within units highlight the need for clear recommendations provided by standardized guidelines.

## Introduction

The routine clinical approach of prophylactic caffeine in neonatal intensive care units (NICUs) has become well-established due to its positive outcomes in both the early and late stages, and it is recommended in both international and national guidelines (1–5). Common recommendations regarding caffeine usage are based on the methodology of the CAP study; caffeine should be administered within the first 72 h to preterm infants at high risk of apnea, with a loading dose of 20 mg/kg and a maintenance dose between 5 and 10 mg/kg (6). However, these guidelines do not provide specific recommendations regarding which preterm infants should receive caffeine treatment, the exact timing of caffeine initiation, dosage adjustments, and the duration of treatment. Instead, they offer a range of options.

In Turkiye, caffeine treatment in premature neonates is outlined in two recommendation papers: one on the prevention and management of bronchopulmonary dysplasia and the other on the management of respiratory distress syndrome and surfactant treatment, both of which have been in 2018. These guidelines recommend the use of caffeine citrate starting from the first day after delivery for infants with a gestational age of <28 weeks. For infants up to 32 weeks, if positive pressure ventilation is required, the decision should be made on an individual basis.

Two globally accepted guidelines, The European Consensus Guidelines on the Management of Respiratory Distress Syndrome and the Specialist Neonatal Respiratory Care for Babies Born Preterm-NICE guideline, both of which were published after our national guidelines, still do not provide specific recommendations. This leads to individual variations in caffeine usage both among and within units. Therefore, optimal dosage adjustments, as well as the timing and course of caffeine treatment, still require further research.

Studies from different countries have examined the prescribing variability in units and emphasized the necessity of standardizing caffeine usage, suggesting that variations in caffeine usage may not yield positive outcomes for the health of preterm infants (7–10). Grainge et al. from the United Kingdom emphasized some variation in practice regarding the timing of caffeine initiation, gestational age cut-off for routine caffeine prescription, and discontinuation (7). Ji et al. focused on the discontinuation timing of caffeine premature infants in the United States (8).

This survey study was planned to investigate the variability in caffeine usage practices. Questions were formulated regarding the patient population, initiation timing, loading and maintenance doses, dose adjustments, and duration of treatment. It was planned to compare the data from our country with studies in the literature showing similar variations.

## Materials and methods

Between February and March 2024, we conducted a prospective online survey. The institutional ethical committee approved the study (KA23/428). In Türkiye, the total number of NICUs including neonatologists on the medical staff is 134 (11). All neonatologists are members of the Turkish Neonatal Society and communication between them occurs via Google Groups platform. The survey was formed with Survey Monkey and the link was sent through an invitation mail in Google Groups. One neonatologist from each unit was requested to respond to the survey on behalf of the unit. It was requested to check multiple choices in case of different opinions within the unit.

The survey encompassed two inquiries regarding personal data four inquiries regarding unit demographics and 15 questions directly addressing caffeine usage. Query topics included caffeine indication for initiation, loading and maintenance dosage, adjustments, discontinuation, and discharge with caffeine treatment. The questions on caffeine usage, detailed in Supplementary, include three questions on caffeine treatment initiation regarding gestational age and indication, two questions on dose and time of loading, four questions on maintenance treatment, five questions on discontinuation, and one question on discharge with caffeine treatment. The 15 questions addressing caffeine usage were designed to include one matrix question, three open-ended questions, one multiple-choice question, and 10 checkbox questions.

## Statistical analysis

A chi-square test was conducted to evaluate the association between gestational age groups and decision criteria. The analysis included all decision-making categories to ensure a comprehensive comparison across gestational age groups. Results indicated a highly significant relationship (*p* < 0.001) between gestational age and NICU preferences, confirming that caffeine therapy decisions vary significantly across gestational age groups.

The methodology involved both statistical analysis and visualization techniques to identify significant patterns and trends. Data was visualized using line and bar plots to examine trends in caffeine initiation preferences across gestational age groups.

The distribution of responses to the matrix question asking which indication caffeine therapy was given for, based on gestational age, was evaluated using descriptive statistics, specifically “variance.” For this purpose, the responses for each gestational week category (<27^6/7^, 28^0–6/7^, 29^0–6/7^, 30^0/7^–31^6/7^, 32^0/7^–33^6/7^, 34^0/7^–36^6/7^, >37^0/7^) were numbered from 1 to 6 based on the frequency of answers.

Intersection Analysis (UpSet Plot) of time intervals for initiation of caffeine therapy and discharge time. The bar plot quantifies the size of intersections, indicating the number of data points shared between specific combinations of intervals. An UpSet plot was used to analyze overlaps and intersections of specific decision categories across time of initiation of caffeine treatment and discharge time. This visualization provided a clear understanding of how categories interact and overlap.

## Results

**Figure 1:**
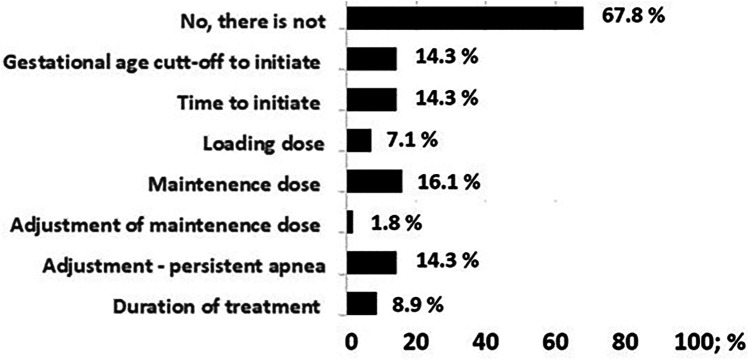
Bar graphic demonstrating the differences in the caffeine treatment protocol among physicians in 56 units with more than one neonatology specialist with decision-making authority.

A total of 74 NICUs responded, accounting for 55% of all neonatology units that include neonatologists on their medical staff. Of the NICUs surveyed, 32 units (43.8%) reported following a standardized protocol. There were 56 units with multiple consultant physicians and 18 of them (32.1%) reported intra-unit variations. The variability in consultant decisions within the units ranged from 16% to 1.8%. The largest difference observed was in the starting dose of the maintenance dose, with a 16% variation. The least variation was observed in the loading dose, treatment duration, and adjustment of the maintenance dose (Figure 1).

**Figure 2:**
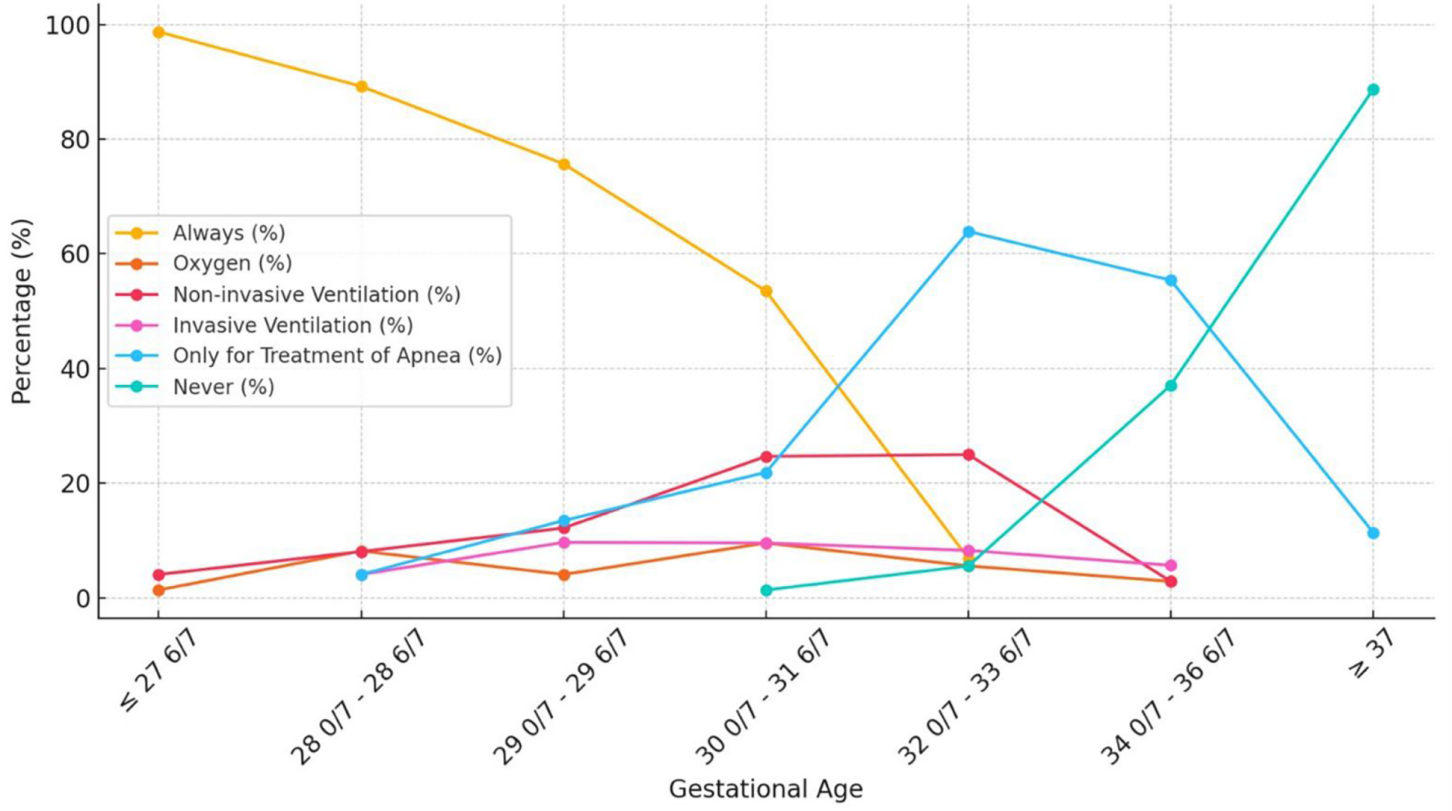
The variability in caffeine practices by gestational age and respiratory support. The variance value for each gestational age group was found as follows; GA < 27^6/7^: 0.163, GA = 28^0–6/7^: 1.047, GA = 29^0–6/7^: 1.348, GA = 30^0/7^–31^6/7^: 2.091, GA = 32^0/7^–33^6/7^: 2.097, GA = 34^0/7^–36^6/7^: 0.828, GA > 37^0/7^: 0.101.

Detailed data of survey is presented in Table 1. The variability in prophylactic caffeine usage based on gestational age (GA) was observed among units for cases not receiving respiratory support: GA < 27^6/7^: *n* = 73; 98.6%, GA = 28^0–6/7^:*n* = 65; 89.0%, GA = 29^0–6/7^:*n* = 55; 75.3%, GA = 30^0/7^–31^6/7^:*n* = 39; 53.4%. 26 (35.1%) units indicated initiating caffeine for any respiratory support between GA = 32/0–33/6. For apnea treatment, 41 (55.4%) units reported using caffeine for late preterm infants, while 8 (11.4%) units reported its use for term infants. Table 2 shows the preferences for starting caffeine in infants regarding to gestational age and respiratory support. Our results show that the greatest variation occurs in gestational age between 30 and 32 weeks. These variations can be visualized in Figure 2. The variance value for each gestational age group was found as follows; GA < 27^6/7^: 0.163, GA = 28^0–6/7^: 1.047, GA = 29^0–6/7^: 1.348, GA = 30^0/7^–31^6/7^: 2.091, GA = 32^0/7^–33^6/7^: 2.097, GA = 34^0/7^–36^6/7^: 0.828, GA > 37^0/7^: 0.101.

**Table 1:** Survey questions and answers of participants regarding the details of caffeine treatment.

Survey question	Survey answer	Answers of participants *n* (%)^a^
For infants <32 weeks of gestation in your unit, for what purposes do you start caffeine treatment?^MC^	Only for prophylactic purposes	62 (82.4)
	Only for therapeutic purposes	–
	Both	13 (17.6)
Do you pay attention to the birth weight threshold of 1,250 grams when deciding on caffeine treatment indication?^MC^	Yes	46 (62.2)
	No	25 (33.8)
	Both approach in unit	3 (4.1)
For the following gestational week intervals, indicate your preference for starting caffeine.^MQ^	Please refer to Figure 1	
What is your preferred time for caffeine loading?^CQ^	0–2 h	46 (62.2)
	3–6 h	18 (24.3)
	7–12 h	8 (10.8)
	13–23 h	8 (10.8)
	24–47 h	11 (14.9)
	48–72 h	2 (2.7)
	>72 h	3 (4.1)
What is your preferred dose for caffeine loading? (As caffeine citrate)^CQ^	20 mg/kg	72 (97.3)
	10 mg/kg	3 (4.0)
	Other	–
What is your preferred starting dose for caffeine maintenance treatment? (As caffeine citrate)^CQ^	5 mg/kg	48 (64.9)
	10 mg/kg	24 (32.3)
	Other^b^	6 (5.3)
What is your preferred dose range for caffeine maintenance treatment?^CQ^	Every 12 h	6 (8.1)
	Every 24 h	72 (97.3)
	Other	–
Do you adjust the maintenance dose in asymptomatic cases?^CQ^	No	35 (47.3)
	Yes, according to the weight change	41 (55.4)
	Other^c^	7 (9.4)
Do you change the dose of caffeine treatment in symptomatic cases?^CQ^	Only mini loading	15 (20.3)
	Mini loading **+** Increase in maintenance dose	30 (40.6)
	Increase in maintenance dose to 10 mg/kg	30 (40.6)
	Increase in maintenance dose by 1–2 mg/kg	12 (16.2)
	Other^d^	3 (4.2)
For infants without apnea and respiratory support, at what PMA do you stop treatment?^OA^	Median (min—max): 34 (32–36)	
For infants without apnea but any respiratory support, at what PMA do you stop treatment?^OA^	Median (min—max): 36 (34–52)	
What is your approach to the use of caffeine treatment after postmenstrual week 36?^CQ^	Independent of apnea, as long as intubation	20 (27.0)
	Independent of apnea, as long as NIV	19 (25.7)
	As long as apnea persists	51 (68.9)
	In the presence of intermittent hypoxia	18 (24.3)
	Caffeine is discontinued after 36th PMA	17 (23.0)
	Other	–
Regardless of the clinical condition, up to which PMA at most would you use caffeine treatment?^OA^	Median (min—max): 40 (36–52)	
How many days do you wait for discharge after discontinuing caffeine?^CQ^	1–4 days	28 (37.9)
	5–7 days	51 (68.9)
	8–10 days	–
What is your opinion on discharge with caffeine treatment?^CQ^	Discharge is not done with caffeine treatment	47 (63.5)
	There were few cases	13 (17.6)
	We have no experience, but acceptable	18 (24.3)
	Other	–

MC, Multiple choice; CQ, Checkbox question; MQ, Matrix question; OA, Open answer; PMA, Postmenstruel age.

^a^ One neonatologist from each unit was requested to respond to the survey on behalf of the unit. It was requested to check multiple choices in case of different opinions within the unit. The “*n*” refers to the total number of given anwers in checkboc and matirx questions.

^b^ 6 mg/kg (*n* = 3), 7 mg/kg (*n* = 2), 8 mg/kg (*n* = 2).

^c^ 1 mg/kg/week to a maximum of 10 mg/kg (*n* = 4), Koch protocol (*n* = 3).

^d^ 20 mg/kg loading dose (*n* = 2), twice daily regimen (*n* = 1).

**Table 2:** **Q**: for the following gestational week intervals please indicate your preference for starting caffeine.^Matrix question.^

Gestational age	Always	The least respiratory support needed to decide for prophylactic treatment			Only for treatment of apnea	Never	*n*
		Oxygen	Non-invasive ventilation	Invasive ventilation			
≤27^6/7^	98.7%	1.4%	4.1%	–	–	–	74
28^0/7^–28^6/7^	89.2%	8.2%	8.1%	4.1%	4.1%	–	74
29^0/7^–29^6/7^	75.7%	4.1%	12.2%	9.7%	13.5%	–	74
30^0/7^–31^6/7^	53.5%	9.6%	24.7%	9.6%	21.9%	1.4%	73
32^0/7^–33^6/7^	6.9%	5.6%	25.0%	8.3%	63.9%	5.6%	72
34^0/7^–36^6/7^	–	2.9%	2.9%	5.7%	55.4%	37.1%	70
≥37	–	–	–	–	11.4%	88.7%	71

Administering caffeine loading dose within the first two hours was reported in 46 (62.2%) units. The starting dose for caffeine maintenance was 5 mg/kg in 48 (64.8%) units, 10 mg/kg in 24 (32.4%) units, and intermediate dose in 6 units. 35 (47.3%) units reported no routine dose adjustment.

Different practices were reported for caffeine dose management in the presence of apnea: mini-loading: *n* = 15; 20.3%, mini-loading and increase in maintenance dose: *n* = 30; 40.6%, increasing maintenance dose to 10 mg/kg: *n* = 30; 40.6%, and increasing maintenance dose: *n* = 12; 16.2%. One unit reported shortening the dose interval.

**Figure 3:**
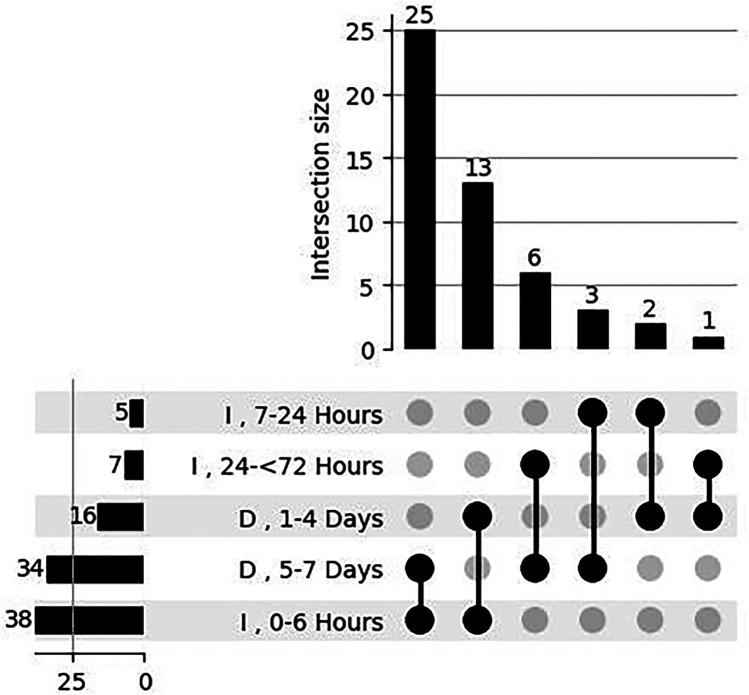
Intersection analysis of time intervals for initiation of caffeine therapy and discharge time. The bar plot quantifies the size of intersections, indicating the number of data points shared between specific combinations of intervals. The intersection matrix at the bottom visually maps these combinations, with vertical lines connecting dots to represent overlapping intervals. On the left, horizontal bars summarize the total count of data points for each individual time interval, offering a clear overview of category sizes. This comprehensive visualization highlights the dominant time intervals and their overlap in caffeine therapy application. I: Initiation, D: Discharge.

For cases without apnea but ongoing respiratory support, caffeine treatment was discontinued between postmenstrual weeks 33–52, and in 14 units, caffeine was not used after postmenstrual week 36. Variability was also observed in discharge timing after treatment cessation; 1–4 days: *n* = 28; 37.8%, 5–7 days: *n* = 51; 68.9%. For neonates without respiratory support, the most common age for caffeine discontinuation is 34 weeks, followed by 36 weeks. However, practices vary widely with some units extending to 40 weeks or beyond. For neonates with respiratory support, caffeine often continues until respiratory support is discontinued (36–40 weeks). There was no correlation between caffeine initiation time and discharge time after discontinuation. The intersection of various time intervals for caffeine therapy, providing insights into both individual and combined interval contributions are given in Figure 3.

## Discussion

Caffeine citrate began to find its place in the care of premature infants with the groundbreaking study by Jacop Aranda in 1977 (12). Initially used for the treatment of premature apnea, caffeine citrate gradually became more commonly used in neonatal intensive care units over the years. Concerns regarding the mechanism of action of caffeine, namely adenosine receptor blockade, led to the initiation of the CAP study as a safety measure, which reported groundbreaking results regarding the benefits of caffeine (6). With the accumulation of these results, the purpose of caffeine therapy evolved. Now, the aim of caffeine therapy is not only to treat apnea but also to prevent it, as well as to reduce bronchopulmonary dysplasia, and achieve positive neurodevelopmental outcomes (13–15). The accumulation of evidence that premature retinopathy, patent ductus arteriosus, acute kidney injury, and inflammation may also be reduced in infants receiving caffeine led to a shift in the perspective on caffeine therapy in neonatology (16–18).

Current guidelines provide varying recommendations for the gestational age threshold at which caffeine therapy should be initiated. The European RDS Consensus guideline recommends caffeine therapy for all premature infants born below 32 weeks gestation receiving positive pressure ventilation, while the United Kingdom guidelines suggest its use for all infants born below 30 weeks gestation (2, 3). The American Academy of Pediatrics guidelines recommend caffeine therapy for all infants born below 28 weeks gestation and for those born between 28 and 32 weeks gestation who are receiving positive pressure ventilation (1). Similarly, our national guidelines recommend caffeine for all preterm infants with a gestational age of <28 weeks and suggest an individualized approach for infants between 28 and 32 weeks requiring positive pressure ventilation. The recommendations endorsed by our association closely align with the AAP guidelines (4, 5). Therefore, indications for caffeine therapy may vary in infants born between 28 and 32 weeks' gestation depending on the guidelines followed.

Our survey indicated that nearly all participating units administered caffeine therapy to infants born before 29 weeks gestation, regardless of respiratory support. Beyond 29 weeks, respiratory support became an increasingly important factor in therapy decisions. The greatest variability in practices was observed between 30 and 34 weeks gestation, particularly at 30–32 weeks, reflecting the complexity of clinical decision-making during this period. This variability highlights a gap in international guidelines, which provide limited recommendations for caffeine therapy in this range, leading to reliance on individual judgment and institutional preferences. Comprehensive, evidence-based guidelines are needed to harmonize practices and improve outcomes for preterm infants. Similarly, Greinge et al. found wide variation in caffeine initiation thresholds among units but did not assess respiratory support, which may limit the applicability of their findings (7).

Caffeine use in moderate and late preterm infants remains debated. Apnea occurs in 20% of infants born at 32–34 weeks and 10% at 34–36 weeks gestation (19, 20). Intermittent hypoxia, associated with adverse outcomes, is also common in these groups (21). While evidence on caffeine therapy for these infants is limited, some studies suggest potential benefits (22–24). Our survey found greater variability in initiating caffeine therapy among moderate preterm infants compared to late preterm infants, with half of the units administering it therapeutically between 32 and 37 weeks gestation.

The favor of early caffeine treatment was first shown by CAP trial subgroup analyses (25). Early treatment, defined as administration within the first 3 days of life, is supported by meta-analyses and systematic reviews showing reduced rates of bronchopulmonary dysplasia, patent ductus arteriosus, periventricular leukomalacia, intraventricular hemorrhage, and severe retinopathy of prematurity, along with improved neurodevelopmental outcomes (2, 26–32). However, the optimal timing within these 3 days remains unclear, and the question of whether “earlier is always better” persists. While very early caffeine use, such as at birth or within 2 h, has shown benefits like improved lung mechanics and reduced ventilation needs, concerns about mesenteric blood flow disturbances remain (33–37). Research comparing timing within the first 72 h has not found significant clinical differences. Notably, our survey is the first to provide detailed data on loading dose timing, revealing that two-thirds of units administer caffeine within the first day, with significant variation, and about one-sixth start it on the second day.

Recent reviews show that high-dose regimens reduce apnea, extubation failure, and BPD without affecting mortality, though evidence on neurodevelopmental outcomes is conflicting (24, 38–45). Current guidelines recommend 20 mg/kg as a loading dose and 5–10 mg/kg for maintenance, but the two-fold range in maintenance dosing highlights uncertainty about the optimal starting dose. While higher maintenance doses improve short-term respiratory outcomes and reduce bronchopulmonary dysplasia, initiating with 10 mg/kg remains limited to clinical research (46–48). Surveys from the United Kingdom and New Zealand, consistent with our findings, reported 20 mg/kg and 5 mg/kg as the most common doses, with one-third of units using a maintenance dose of 10 mg/kg (7, 10).

Dose adjustment in asymptomatic patients lacks clear guidelines. Dose adjustment in asymptomatic patients is another issue of debate. The CAP study protocol, which starts with a 5 mg/kg maintenance dose and increases weekly based on weight, is an acceptable minimum approach. Some studies have used intermediate doses (49, 50). A United Kingdom survey reported that 86% of units regularly optimize caffeine doses based on weight, while our survey found that 55% of units adjust doses weekly. The lack of clear guidance in international guidelines may explain why dose adjustments based on weight are not performed in half of the units.

As caffeine therapy's role has expanded from treating apnea to providing multisystemic benefits, studies have focused on the concentration-effect relationship. Serum caffeine levels between 5 and 20 mg/dl are considered sufficient for apnea prevention, with higher levels (15–20 mg/dl) showing a positive impact on chronic lung disease (13, 51, 52). While pharmacokinetic data can optimize dosing, they are not always available in routine practice. Studies have considered factors like weight gain and liver metabolism in neonates to determine the appropriate serum level (53–57). Koch et al. recommend gradually escalating the dose to 8 mg/kg from the 5th week onward, a protocol endorsed by the latest European RDS guidelines (2, 57). However, this approach is not widely adopted, with only two units reporting its use.

There is also uncertainty about how to increase the dose in cases with ongoing apnea of prematurity. The CAP trial indicated that the maintenance dose was increased to 10 mg/kg. However, there is evidence that a small loading dose together with an increased maintenance dose is successful in decreasing apnea (58). Gray et al. stated that the maintenance dose was increased in the majority of units (10). Yet, we also observed that just the increase of the maintenance dose or mini loading plus an increase of the maintenance dose wer equally preferred within units.

While the benefits of caffeine therapy are well-established, it is unclear when to discontinue it relative to the baby's discharge. Neonatologists typically require an “event-free” period after stopping caffeine before discharge, but there is no consensus on how long this apnea-free period should be. American Academy of Pediatrics suggests stopping caffeine treatment when the corrected age of the baby reaches 33–34 weeks and they have been free from apnea/positive pressure for one week, which is later while United Kingdom guidelines recommendation includes the farthest outcome (1, 3). In the United Kingdom, 34% of units would stop caffeine at 34 weeks, regardless of respiratory support, while 11% would continue if respiratory support was needed (7). A similar variability was observed in a study by Ji et al. The authors emphasized that respiratory support at the time of discontinuation was common but variable, with 0%–57% of infants receiving positive airway pressure at caffeine discontinuation by site (8). Ducrocq et al. reported that postmenstrual age for discontinuation ranged from 32 to 40 weeks in France (9). Our survey showed a trend of prolonged caffeine treatment based on respiratory support needs, with similar variations observed in caffeine use beyond 36 weeks postmenstrual age. The ongoing apnea or intermittent hypoxemia in premature infants after 34 weeks, along with the benefits of caffeine, forms the clinical approach to this undefined guideline issue (26, 59–61).

The safe serum level considered for discharge after caffeine therapy is <5 mg/dl, which indicates subtherapeutic levels. As can be understood, the time required for the serum level to drop to this level is dose-dependent due to the long half-life (62). Chung et al. showed that the proportion of cases with serum levels higher than the subtherapeutic level after discontinuation of caffeine therapy at ≥5 mg/kg/dose was approximately 50% between 5 and 7 days and around 25% between 8 and 10 days (63). The lack of guidelines results in variable durations for discharge after cessation of caffeine therapy (10).

While our survey garnered responses from 55% of neonatal units, it's significant that the majority of these responses originated from units with long-standing staff. The fact that only one doctor exists in some units may limit the accuracy of the data. Additionally, the response rate is comparable to other multicenter studies guided by the Turkish Neonatal Society suggesting that these responses likely reflect the sentiments of neonatologists across Türkiye (11, 64). Notably, our response rate mirrors that of the study by Grainge et al., a survey conducted in the United Kingdom (7).

One noteworthy aspect of our survey is the documentation of intra-unit preferences. The fact that our national guidelines were published in 2018, followed by the update of the NICE guideline in 2019 and the European Consensus Guidelines on the Management of Respiratory Distress Syndrome in 2022, indicates that neonatologists have clearly followed global guidelines. The significant differences in caffeine usage preferences between units may stem from the lack of clarity in the recommendations of the updated international guidelines. Unlike the United Kingdom survey, which lacked an acknowledgment of physician bias, our survey sheds light on this issue, adding an important dimension to the discussion. The other strength of the current survey is that caffeine use in infants greater than 32 weeks was evaluated. It's important to highlight that there were variations in multiple aspects of caffeine usage.

In conclusion, our survey revealed discrepancies concerning the timing of both caffeine initiation and cessation, gestational age threshold for routine caffeine commencement, and as well as dose adjustment. Intra-unit variability other than national variations underscores the necessity of more focused guidelines. These findings emphasize significant disparities in caffeine utilization across neonatal units in Türkiye.

## Data Availability

The raw data supporting the conclusions of this article will be made available by the authors, without undue reservation.
